# Diseases of the Teeth in the International Medical Congress.—A General Treatment of Irregularities

**Published:** 1882-01

**Authors:** Walter H. Coffin


### AMERICAN JOURNAL

OF

### DENTAL SCIENCE.

Vol. XV. THIRD SERIES—JANUARY, 1882. No. 9.

### ARTICLE I.

*Diseases of the Teeth in the International Medical Congress*

### A GENERALIZED TREATMENT OF IRREGULARITIES.

Mr. Walter H. Coffin read a paper on the above subject. On any case of irregularity presenting for treatment, he said, the practical question would be, What is the best to do, and what is the best way to do it? A classification and analysis, in even their infinite variety, of a sufficient number of instances, and the results of their treatment by every possible means, should afford at least an approximate answer to these, and the no less important questions—What not to do, and how to avoid doing it? The few models shown had been selected, as fairly representative, from several thousand, recording the attempted treatment of perhaps a larger proportion than usual of the ordinary irregularities met with in an average practice, and illustrating the evolution, within certain limits, of an almost generalised method. Classifying any large series most conveniently by the mechanical exigencies of each case, in certain of them extraction of possibly sound teeth might, of course, be necessary, though these were less numerous than usually imagined. A large class, uncomplicated by crowding, admitted of direct and immediate correction by suitable means. Of the remainder, the majority were cases, of every variety, in which the teeth—not really too large or numerous for the jaw they might symmetrically occupy—were by some chance of their eruption irregularly disposed, interlocked, and crowded. Of these it might be affirmed that rectification necessitated the movement of many teeth or all, and an altered shape or outline of the dental arch. This class presented the greatest difficulty in regulating by the usual way, especially with a rigid plate; but in the most intricate or the simpliet of them, the permissive control of the general tendency of movement during regulation, reduced their successful treatment to comparative ease and certainty. This mechanical anticipation of favorable conditions might be illustrated by assuming an incisor to be moved in a crowded arch by any means applied by a plate rigidly embracing the bicuspids and canines, when a certain force in a certain time might complete the operation; but were the plate either abolished or its symmetrical halves partly independent and free to move relatively in the plane of the arch, less time and force would suffice; and, furthermore, if its halves tended but slightly to separate by an elastic spring reaction, many cases would require very much less time and force to be exerted on the tooth. The action thus stated was observed for the first time by a singular accident. Soon after the introduction of vulcanite, the author's father was employing a plate of that material to move an incisor by the swelling of wool. Successive increments of force were resisted until not only was it suddenly in position, but the other front teeth were found slightly separated where previously in overlapping contact; the wool (being nearly on the median line,) by lateral expansion, having split the plate down the centre. In this instance, as would often be the case, previous "expansion of the arch" by the means usually applied was certainly not indicated, and therefore not resorted to, although just the slight amount of spreading required was prevented by the rigid construction of the plate. A conviction of this led to a particular method of treating various irregularities, which, as anticipating changes come to them—usually expansive—had been called somewhat indefinitely an "expansion treatment;" and the adoption of which has been abundantly justified by experience. The "expansion plate," whether used for direct expansion or not, was of extreme simplicity, while of complex regulating action; comprising a means—easily embodied in any plate—of conveniently permitting or assisting (instead of hindering or preventing) during regulation, the inevitable changes of the arch naturally accompanying it; and supplementing ordinary expedients with an expensive characteristic. Its distinguishing function depended on the principle of permitting a relative motion, or maintaining a particular controllable reaction between two semi-independent parts, usually its symmetrical halves. Difficulties attended the first realization of this condition; but it was found that a wire spring of certain form, if a constructive part of the plate, would itself meet all requirements.

The general form might be described as a rather thin vulcanite plate, capping and clasping some or all of the bicuspids and molars, and fitting the lingual surfaces of anterior teeth; but divided completely along the median line into two distinct halves, which were connected by a slight steel wire, so disposed that, while guiding and limiting their relative motion, its tension exerted between them might be perfectly determined and varied in direction and magnitude. When necessary in such a plate to establish the spring reaction, a surprisingly small, almost imperperceptible *stress*, even so distributed, and against a widespread resistance, if continuously maintained and suitably applied, sufficed to produce any degree of motion desired. The perfection of the model must be insisted upon. The best impressions had been obtained with the preparations of gutta-percha or ballata gum, no other material affording with ease the absolute fit essential for a split plate. A

delicate and elastic vulcanite plate from a gutta-percha impression would generally spring over the teeth with so absolute a fit, that its removal might even be embarrassing; but until divided its insertion was not usually attempted.

Trials of the metals and their alloys proved the superiority for springs of apparently so undesirable a metal as steel. The almost insuperable difficulty of satisfactorily tempering bent soft steel without deformation of shape, was obviated by the use of pianoforte wire, possessing very uniform texture, temper permitting it to be fashioned and used without heating, and a surface hardness and burnish which greatly tended to its preservation. A diameter of between three and four hundredths of an inch (or about 0.035 inch) was most suitable, as of this a convenient length of from one to two and a half inches exerted an appropriate tension in average cases. The extremities being buried rigidly in the vulcanite, the uncovered and active portion of the wire, emerging from selected points in the alveolar region, should be entirely on the lingual side of the plate, nicely fitting, but free to move upon its surface. The wire between its attachments might be in a simple curve, when, for localised action (as exclusively posterior expansion,) it would urge a relative motion or rotation about some point; but that very kind of motion might be established, there must be one or more reversals of curvature, by either a single couple of opposite curves, or any number of alternately contrary ones (preferably *odd*) approximately balanced, and as large and symmetrical as possible. A serviceable form for an upper general expander was a three or five-curve serpentine figure, like a rounded capital W. The spring being shaped to fit as nearly as possible the palatal surface of an upper model, or the lingual surface of a lower, had its ends for half an inch (without being softened) slightly flattened and roughened, and so bent towards the model as to raise it uniformly from the surface to a distance of about the desired thickness of the plate, and the portion to be inserted in the rubber tinned or coated with common solder. To several points upon its exposed part, short ends, or loops of binding wire, were twisted to better secure it in the plaster investment. When in shape it must be free from tension, and attached in the vulcanization, to the plate, which was made *entire* and afterwards divided. The plate being modelled in wax, the spring was placed on the surface, with its ends buried within, and when removed by the counterpart, protected from the rubber by tin foil before packing. Finished in the usual manner, entire, the plate was divided with a fine saw, the edges and corners of the cleft being well rounded and smoothed. This, with care, might be done without imparting tension or twist to the spring, which was important. The plate should be inserted and worn in the mouth without tension for a day or two, to first eliminate causes of irritation not due to its expansive action, and sooner induce toleration of its presence. Any expansive force required might then be established, and its right direction secured, by stretching the two halves with a slight exaggeration into the relative positions towards which it was desired they should tend to move. This, however, might require correction and modification by observing its effects in the mouth. The amount of stress, which was not so conveniently diminished as increased, should be small at first, especially in plates whose expansive function might be simply permissive or auxiliary to other regulating action. It might safely be entrusted to the patient (even quite young) to frequently remove, clean, and replace, after duly cautioning, without fear of disturbing its adjustment. The experience gained of steel wire had led to its almost exclusive adoption for ordinary regulating purposes, as spring levers acted directly on the teeth, for pulling, pushing, or rotating; and, being permanently fixed to the plate, their convenience, adjustability, and many adaptations, were remarkable. Combined with a split plate they were found to replace with advantage, screws, inclined planes, wedges, levers, and ligatures, in many of their local uses, and, moreover, were practicable where nothing else could be applied. In fact, by the gradual simplification of means, and the elimination of uncertain devices, a delicate split plate, with perhaps one or two short lengths of small wire closely fitting its surface, was often now to those who employed it, the representative of the wondrous combinations of nearly all the known "mechanical powers" that once exercised their ingenuity. Where in simple cases a plate was unnecessary, with an inch or two of steel wire and elastic rubber tube, efficient expedients might frequently be improvised.

When direct expansion of the arch was indicated, the thin, spring reaction plate, fitting closely the palate, teeth, and tissues covering the alveoli, and not filling the mouth or impeding any of its functions, would, with the minimum of trouble and attention, effect any desired degree of that spreading and moulding of the mouth which it was well-known might, to a surprising extent, be rapidly and almost painlessly produced.

Among the uses of direct expansion, its interesting application to the operative treatment of caries, and to slightly overlapping incisors, might be discussed together. In young mouths where interstitial decay of incisors closely in contact almost defied successful treatment, any operation attempted would, of course, be facilitated by gaining ever so small a space between them. Paradoxical as it might seem, it was actually less painful and troublesome to secure ample space between all the front teeth at once, than to wedge two of them apart in the ordinary way, with the advantage of easily maintaining their separation without irritation during any course of treatment. A plate exerting anterior expansious would rapidly—and almost painlessly—separate the incisors, and by a suitable adjustment of the bearing surfaces, might readily be caused to distribute spaces equally between all the front teeth. If the incisors were just overlapping, slight expansion, with suitable disposition of the bearing surface afterwards, usually permitting the natural action of the lips to straighten them. Expansion, where posterior teeth wrongly articulated, presented little difficulty when the conditions were symmetrical on both sides; when otherwise, or confined to one side only, a differential or even a unilateral effect might be produced, according to the number and kind of teeth the action of the plate might be distributed or concentrated upon, and the arrangement of its articulating surfaces. Lastly, the obvious application of the divided spring plate to narrow misshaped mouths with high contracted roofs, unfortunately also presented considerable difficulty. These cases admitted of great improvement (as illustrated by models exhibited) where circumstances were favorable for expansion; as when the bite could be either kept normal or made so, and the jaw was not really so concentrated as the dental arch made it appear; for such the split reaction plate was peculiarly adapted and unsurpassable, if treated with caution.

While expansion was most readily performed in young mouths, especially under sixteen, the only limitation of age would seem to be the diminished power of restoring fixity after movement, which must be inferred of advanced age, though in a case of considerable expansion at forty-five, the teeth became perfectly firm afterwards. It was hardly necessary to dwell on the desirability of the greatest possible simplicity of regulating devices, in principle and details; and the self-maintaining adjustability of plates, that they might be as independent as possible of any control by the patient, whose co-operation should be confined to their perfect cleanliness only, and the health and comfort of the mouth. In final justification, the advocates of the method appealed to their records of results, which—of whatever real importance or value—would have been difficult or impossible to otherwise attain; and had ventured at such length to detail their procedure, for confirmation or criticism by others.

This paper was illustrated by more than 500 old regulating plates which had been actually used; about 400 being "expansion plates," upper and lower, symmetrically and unsymmetrically divided, of which nearly 200 were "simple expanders," some 200 embodying other regulating devices with "expansion;" the remainder showing the application of piano-forte wire in ordinary plates for general regulating purposes. There were also specimens, with demonstrations, showing in different stages, details of their mode of construction. The models exhibited in the Museum of the Congress, at Burlington House, of forty typical cases, showing by three or more casts of each case, the condition before, during, and after treatment, were classified as illustrating—

1. Expansion auxiliary to ordinary regulating (*a*) in simple crowding, (*b*) for rotation and alignment.

2. General expansion, (*a*) for operative treatment of caries, (*b*) for misarticulation, (*c*) *versus* extraction of misplaced teeth, (*d*) for prominent incisors, (*e*) for contracted, narrow, or misshaped arch.

3. Applications of steel wire to every kind of ordinary regulating, (*a*) alone, without plate or accessories, for alignment or rotation (*b*) combined with elastic ligatures, (*c*) with an ordinary plate for moving, shortening, and rotating teeth.

4. Combinations of the above.

---

### THE CAUSES OF IRREGULARITIES OF POSITION OF TEETH.

The Secretary then read a paper on the above subject by Dr. Thomas Brian Gunning, of New York, who was himself prevented from attending. The writer advocated the regulation of teeth almost as soon as they appeared, long prior to the time at which the profession in this country are accustomed to interfere with them. The relation of the jaws to the teeth and their alveolar processes needed to be explained, since it was not only imperfectly understood but even misrepresented, it being asserted that the jaws were developed independently of the teeth and their alveolar processes, and that the teeth might be removed without affecting the jaws. He pointed out that in the natural evolution of teeth, the upper jaw was supported by the lip, the nipple being held against the roof of the mouth by the stronger and more active lower lip, assisted by the less developed jaw ; that tended to keep the lower jaw back in its proper position, with the upper jaw projecting. He then went on to describe the familiar cases of backward elongation and the successive addition of the first, second, and third permanent molars to the back of the jaw. He altogether impugned the idea that some portions of the jaw grew independently of the teeth, whilst only portions directly dependent on the teeth were the alveolar borders. As the lower permanent teeth generally appeared before the upper ones so the expansion of the lower jaw was somewhat earlier than that of the upper. In the time between the completion of the first set and the appearance of the six-year old molars, the lower jaw might grow so that its incisors struck on the edge of the upper front teeth, and in order to rest on the back teeth the muscles in certain cases habitually held the edge of the front teeth forward, or the jaw might be held out of its natural position from the too early loss of the infant incisors, allowing the permanent ones to grow up in such a way as to be uncomfortable. When the infant teeth remain too long, the permanent teeth might be kept out of range, and the jaw habitually held out of its natural position. This should be corrected. The forward action of the lower jaw might also commence through the tenderness of the front teeth of the other jaw ; or if the side teeth were painful the muscles might hold the jaw out of centre or it might grow so. Sometimes surgeons were deceived in cases of this kind ; he once had a patient who had been treated for fracture of the jaw through a fall, but in reality there was only this habitual malposition of the teeth. As to extraction, he was of opinion that even if the teeth as a whole were too large for the jaw they ought to be kept in, to encourage the growth of the jaw, so long as that could be promoted by this means, and then, and only then, such of them extracted as could be best spared to make room for the others. In conclusion, Dr. Gunning complained that, although odontological practitioners enjoyed at this time the greatest facilities in their various appliances, whilst their works of reference were so numerous, he failed to find on a careful scrutiny anything which could give him the assurance that they regulated teeth better than men in Germany and other parts of Europe did fifty years ago.

In the discussion which ensued upon these papers, Dr. Rosenthal, of Liege, stated that he had long employed Mr. Coffin's system with great satisfaction, had been surprised at its simplicity and efficiency, and was gratified at the ease of producing results which he had thought impossible.

Mr. Cunningham, of Wizbech, confessed to the great assistance he had derived from Mr. Coffin's extension plates, the contraction and use of which, however, depended so greatly on details, that he attributed his own success largely to the personal instruction and advice of Mr. Coffin. He therefore regretted that the paper omitted many important points, as, for instance, the description of the use of gutta percha, which, though many thought they knew all about it, he found by Mr. Coffin's peculiar method had revolutionized the taking of impressions. He thought that all who really tried this method of expansion—which deserved wider recognition—would find, as he had found, many unexpected and useful applications of the split plate.

The President, Mr. Edwin Saunders, recited a case which he said was now puzzling him. The patient, a boy, sixteen years of age, who had just cut the right central incisor immediately in front of the left, so precisely one over the other that they both occupied apparently the same position in relation to a line drawn straight from the root of each tooth, being exactly in the same plane. He had made a plate in order to bring it into its proper position, but the difficulty was that he could not get the patient to use the plate continuously.

Mr. Coffin then briefly replied to the remarks made on his paper.

---

### THE ORIGIN AND TREATMENT OF CERTAIN IRREGULARITIES OF THE TEETH.

BY OAKLEY COLES.

---

In my classification of the deformities of the upper jaw, I have endeavored to establish the theory that a large number of cases of irregularities of the teeth are due to a malformation of the jaw rather than to a mere displacement of the dental organs. In other words, I have asserted that the arrangement of the teeth is but the expression of a profound morphological change. In the continuance of my investigations in this direction, I desire to call attention on the present occasion to the variety of the brachoid jaw in which the region of the bicuspid teeth is the seat of lateral compression. In order to render the argument clear by which I have arrived at present conclusions, it will be necessary to refer briefly to the anatomy and growth of the parts which seem to be involved in the development of the deformity under consideration.

It is a fact that will scarcely demand proof or verification, that at birth all upper jaws are normal in size and outline. At this date the antra are little more than rudimentary, and occupy but a small space between the base of the alveoli and the floor of the orbit. The intermaxillary articulation is generally normal in character, and the position of the lower jaw in relation to the upper rarely affords any evidence of the changes to which they will afterwards be subjected. Whatever may be the influence of heredity, such influence finds expression during the period of growth, rather than that of development, and structural and functional derangements are manifested and intensified chiefly at a date subsequent to the shedding of the deciduous teeth. In the perfectly healthy subject, the face and jaws will have arrived at a perfect state of development at about the age of puberty. The features of the face will have become permanently marked out by the gradual growth downwards and forwards of the upper maxillary bones and adjacent structures. The chief factor in the production of these results is the growth of the body and wings of the sphenoid. By the agency of this bone the intermaxillary is carried forward and space provided posteriorly for the increased length of the true maxillary process. The two glenoid cavities become more widely separated from each other, and the direction of the long axis at their articular surfaces, and the corresponding condyloid processes of the lower jaw, becomes subjected to important modifications. During the time that these changes are taking place, the antra will have become considerably increased in size by the lengthening in a vertical direction of this portion of the upper maxillæ. Although the maxillary sinus may vary in capacity from one to eight fluid drachms, it should still physiologically bear a definite relationship to the facial angle. To compensate for the advanced position of the maxillary bones, the palatid bones grow in such directions as to fill up the gap that would otherwise exist between the posterior extremity of the dental arch and the pterygoid processes of the sphenoid.

If all the parts involved in the formation of the upper and lower jaws have grown in proper proportion to each other, the teeth will only be subjected to such forms of irregularity as may arise from some purely mechanical candition confined to the normal area of the dental arch. If, on the other hand, the jaws themselves have been subjected to any irregularity or inequality of growth, the entire dental arch will be the seat of well-marked deformity, and the teeth themselves be but an integral and nor independent element in the production of the conditions we are discussing; or, to put the case in another way, the teeth are in the one instance the cause of the dental irregularity, whilst in the other, they only give expression to a general maxillary deformity in which they are involved.

For the purpose of the present paper I now wish to call attention to the form of brachoid jaw in which the biscupid region gives evidence of lateral compression rather than of lateral expansion. In such a case the six front teeth are crowded together, whilst the second bicuspids are implanted with, in a severe example, but a space of five milimetres between them in the transverse direction. The first bicuspids, although inside the dental arch, are not so seriously disturbed. If the deformity has been allowed to progress unchecked until twenty-one years of age, the grinding surfaces of the bicuspid teeth are frequently on a lower horizontal plane than the remaining dental organs of the upper jaw. Or, in other words, in the case of the four bicuspids, we may say that the bite is raised. The external alveolar wall will show compression corresponding to the teeth as measured from side to side, and in a well-marked case pass vertically upwards, or even outwards towards the level of the floor of the antrum. In many instances the lower jaw will be of unusual dimensions, not merely in comparison with the upper maxilla, but also in comparison with a normally developed jaw of the same age. The interdental articulation is, of course, destroyed, but beyond this the horizontal rami and body are usually thickened, and at both the alveolar and inferior borders present a curious thickening and rounding. The inferior dental arch passes outside the superior dental arch at all points. The mental outline is almost rectangular, whilst the outline of the ascending and horizontal rami is generally obtuse-angled. The soft palate is generally short, as measured from the posterior wall of the pharynx, the vertex of the hard palate is apparently above the normal plane, and the tonsils are almost in every case considerably enlarged.

Externally the face presents certain well marked and characteristic appearances. There is a peculiar flatness and squareness from the root of the nose to below the mental eminence, which I can only describe in the language of an artist, by saying that there is an absence of drawing in these parts. The lines produced by the muscles passing from the superior border and angle of the orbicularis-oris are not as strongly indicated as they should be, and the face generally is noticeable as being devoid of those lines which are generally regarded as myological landmarks. The nose is usually small, whilst the forehead, with the remaining frontal outlines, gives a good facial angle.

The cause which I venture to suggest as an explanation of the origin of this deformity is the irregular and excessive growth of the external wall of the antrum on either side. If we remember the changes that occur in the lengthening of the face downwards and forwards, it will not be difficult to understand that an undue rate of growth of the external wall of the antrum would have the effect of pushing that portion of the alveolar arch indicated as the bicuspid region, not merely downwards but also inwards towards the median line of the hard plate. From the fact that the transverse measurement of the second bicuspids normally corresponds with a similar measurement between the posterior deciduous molars, it is manifest that in the cases under consideration where we find the second bicuspid within five millimetres of each other, this region must not only have failed to preserve the outline presented in the infantile arch, but must also have been subjected to very considerable mechanical force during that period of growth in which the antra have become developed to their normal size.

I have before alluded to the influence exercised by the body and great wings of the sphenoid, and to the operation of the latter in carrying back the condyloid cavities, and with them the condyles of the lower jaw. To this we must, I think, attribute its abnormal size. Whilst from the evidence afforded by the texture of the hair, teeth, and nails, as well as the enlarged tonsils, we may assume that strumous condition of the constitution, which is generally held to explain the imperfect development and irregular growth of the cranial and facial bones. Professor Humphrey has observed that the prominence of the forehead is proportionate to that of the chin, and that the antra in negroes are small and bear a definite relation in size with the angle of the face. It is, therefore, not unreasonable to infer that in the cases we are discussing in which the frontal bones are vertical and the mental region of the lower jaw correspondingly, if not abnormally, developed, the antra should be abnormally large. My clinical observations do not at present permit me to discuss the relationship between the enlarged tonsils and the increased capacity of the maxillary sinues, but I trust we have present with us on this occasion those who may be able to contribute to our knowledge on this importont point.

I have elsewhere endeavored to show that the prominence or diminished size of the intermaxillary region is due either to the increased or diminished force exercised through the vomer by the body of the sphenoid, and a case under the care of my friend Mr. Rose, of double hairlip and cleft palate, that had remained unoperated upon until the patient was thirty-five years of age, will afford corroboration of the soundness of this theory.

The diagrams show the long and well-arched nose, and the intermaxillary bones carried forward with the rest of the nasal septum, and thus afford very strong evidence in favor of the assertion that intermaxillary prognathism is the product of excessive growth of the sphenoid operating through the vomer. In the museum of the Royal College of Surgeons ( Teratological Section, 238, ) there may be seen an interesting specimen illustrating the peculiar form of the walls of the antrum and upper maxillary outline, that I believe we may find in the compressed brachoid arch, as well as the enlargement of the lower jaw to which I already referred. In a paper read before the Royal Society of Edinburgh some twelve years ago by Dr. Smith, some inter-bicuspid measurements were given of a series of cases of congenital cleft palate, and the author of that communication attributes the lateral compression in such instances to "a misdirection of growth dependent upon the absence of the mesial structures, while the superior maxilla is becoming, as the age advances, elongated downwards by the expansion of the antrum." At that time I dissented from such a conclusion, but my later investigations have induced me to think that Dr. Smith was right and I was wrong; I gladly avail myself of this opportunity of saying so, and it will be seen that I have adopted the same explanation for the origin of the compressed brachoid arch.

Time will not permit me to enter at any length into the question of treatment, but I would submit the following points:

1. That if expansion is tried, it should be expansion of the jaw with the teeth *in situ* in the first instance, and regulation of the teeth individually as a subsequent operation, rather than expansion of the *dental* arch by pressure applied to the teeth and their alveoli.

2. And next the desirability of extracting the teeth that are out of position and then restoring the contour of the arch by expansion. This treatment of course applies only to the more severe cases.

I am induced to bring this point forward for discussion as there seems to me a danger that we may in the pride of our professional skill, submit our patients to greater pain and risk by saving teeth than by extracting them.—*British Journal of Dental Science.*

